# Supplementary material for: Blood-brain barrier disruption defines the extracellular metabolome of live human high-grade gliomas
Source: Commun Biol. 2023 Jun 20;6:653. doi: 10.1038/s42003-023-05035-2 (PMC10281947; doi:10.1038/s42003-023-05035-2)
Supplement: Supplementary file 3 — Description of Additional Supplementary Files [file 42003_2023_5035_MOESM3_ESM.pdf]

Description of Additional Supplementary Files

File name: Supplementary Data

Description: Full data provided for all patients, including raw and normalized data from Metabolon. Separate tab includes 162 metabolites utilized for analyses in the paper (named metabolites present in at least 90% of catheters). .gmx library utilized for GSEA is also provided. Bloody versus clean CSF (pooled) and paired raw data are also provided.
