# Supplementary material for: Blood-brain barrier disruption defines the extracellular metabolome of live human high-grade gliomas
Source: Commun Biol. 2023 Jun 20;6:653. doi: 10.1038/s42003-023-05035-2 (PMC10281947; doi:10.1038/s42003-023-05035-2)
Supplement: Supplementary file 5 — Reporting Summary [file 42003_2023_5035_MOESM5_ESM.pdf]

nature portfolio

Corresponding author(s): Terry C. Burns MD PhD

Last updated by author(s): 2023/05/28

Reporting Summary

Nature Portfolio wishes to improve the reproducibility of the work that we publish. This form provides structure for consistency and transparency in reporting. For further information on Nature Portfolio policies, see our Editorial Policies and the Editorial Policy Checklist.

Statistics

For all statistical analyses, confirm that the following items are present in the figure legend, table legend, main text, or Methods section.

n/a	Confirmed	
☐	☒	The exact sample size (*n*) for each experimental group/condition, given as a discrete number and unit of measurement
☐	☒	A statement on whether measurements were taken from distinct samples or whether the same sample was measured repeatedly
☐	☒	The statistical test(s) used AND whether they are one- or two-sided<br>*Only common tests should be described solely by name; describe more complex techniques in the Methods section.*
☒	☐	A description of all covariates tested
☐	☒	A description of any assumptions or corrections, such as tests of normality and adjustment for multiple comparisons
☒	☐	A full description of the statistical parameters including central tendency (e.g. means) or other basic estimates (e.g. regression coefficient) AND variation (e.g. standard deviation) or associated estimates of uncertainty (e.g. confidence intervals)
☐	☒	For null hypothesis testing, the test statistic (e.g. *F*, *t*, *r*) with confidence intervals, effect sizes, degrees of freedom and *P* value noted<br>*Give P values as exact values whenever suitable.*
☒	☐	For Bayesian analysis, information on the choice of priors and Markov chain Monte Carlo settings
☒	☐	For hierarchical and complex designs, identification of the appropriate level for tests and full reporting of outcomes
☒	☐	Estimates of effect sizes (e.g. Cohen's *d*, Pearson's *r*), indicating how they were calculated

Our web collection on statistics for biologists contains articles on many of the points above.

Software and code

Policy information about availability of computer code

Data collection	No softwares were utilized for data collection. Data were obtained from Metabolon or the Mayo Clinic Metabolomics Core and maintained on an internal server.
Data analysis	MetaboAnalyst 5.0 was utilized for creating of Spearman correlation heat map and hierarchical clustering heat maps. Gene Set Enrichment Analysis 4.1.0 was repurposed for metabolite set analysis (enrichment analyses). GraphPad Prism 9.1 was utilized for all other analyses.

For manuscripts utilizing custom algorithms or software that are central to the research but not yet described in published literature, software must be made available to editors and reviewers. We strongly encourage code deposition in a community repository (e.g. GitHub). See the Nature Portfolio guidelines for submitting code & software for further information.

Data

Policy information about availability of data

All manuscripts must include a data availability statement. This statement should provide the following information, where applicable:

- Accession codes, unique identifiers, or web links for publicly available datasets
- A description of any restrictions on data availability
- For clinical datasets or third party data, please ensure that the statement adheres to our policy

All data for the study are available as supplementary data, including raw and normalised data.

Human research participants

Policy information about studies involving human research participants and Sex and Gender in Research.

Reporting on sex and gender	The impact of sex on the glioma metabolome was not evaluated as a covariate due to the relatively small sample size of the study. Of the 14 neurosurgical patients in the study, nine patients were males and five were females. These data are provided in Supplementary Table 1.
Population characteristics	The age range in the population was from the early 20s to late 70s; sex and age range for each patient are provided in Supplementary Table 1. All patients were diagnosed with a glioma, including 1 grade 2 oligodendroglioma, 3 grade 3 oligodendrogliomas, 1 IDH-mutant grade 3gemistocystic astrocytoma, 3 IDH-mutant grade 4 astrocytomas, 1 IDH-wild type H3K27M mutated grade 4 astrocytoma, 1 molecular glioblastoma, and 4 glioblastomas (including one patient who underwent microdialysis for both their primary and repeat surgeries). All tumors were characterized according to the CNS WHO 2021 criteria. Four of the 15 surgeries were for recurrent gliomas. All other characteristics can be found in Table 1.
Recruitment	Patients were recruited from Mayo Clinic's neurosurgical oncology clinic when they were undergoing evaluation for a known or suspected high-grade glioma. All patients were undergoing clinically-indicated resection for their lesion.
Ethics oversight	Mayo Clinic Institutional Review Board

Note that full information on the approval of the study protocol must also be provided in the manuscript.

Field-specific reporting

Please select the one below that is the best fit for your research. If you are not sure, read the appropriate sections before making your selection.

☒ Life sciences ☐ Behavioural & social sciences ☐ Ecological, evolutionary & environmental sciences

For a reference copy of the document with all sections, see nature.com/documents/nr-reporting-summary-flat.pdf

Life sciences study design

All studies must disclose on these points even when the disclosure is negative.

Sample size	No specific sample sizes were chosen for this study as this was originally designed as a safety and feasibility study. Samples were initially analyzed in a first cohort of five patients and then evaluated for reproducibility based on the amount of new samples available in a second cohort of patients. Sample size was dependent on the amount of eligible patients willing to participate in this study.
Data exclusions	Analyses were based on metabolites present in at least 90% of microdialysate catheters to ensure reproducibility of results based on detected metabolites. No other data were excluded from the study.
Replication	Results were first identified in a first cohort of patient and then evaluated in a second independent cohort of patients. The reproducibility of results from the paired bloody-versus-clean CSF analyses was evaluated using an independent batch of pooled bloody-versus-clean CSF samples.
Randomization	No randomization was performed as there was only one group of patients in the study.
Blinding	No blinding was performed for the study as the patient's diagnosis was known for clinical purposes and for determination of what groups to compare to one another.

Reporting for specific materials, systems and methods

We require information from authors about some types of materials, experimental systems and methods used in many studies. Here, indicate whether each material, system or method listed is relevant to your study. If you are not sure if a list item applies to your research, read the appropriate section before selecting a response.

Materials & experimental systems

n/a	Involved in the study
☒	☐ Antibodies
☒	☐ Eukaryotic cell lines
☒	☐ Palaeontology and archaeology
☒	☐ Animals and other organisms
☐	☒ Clinical data
☒	☐ Dual use research of concern

Methods

n/a	Involved in the study
☒	☐ ChIP-seq
☒	☐ Flow cytometry
☒	☐ MRI-based neuroimaging

Clinical data

Policy information about clinical studies

All manuscripts should comply with the ICMJE guidelines for publication of clinical research and a completed CONSORT checklist must be included with all submissions.

Clinical trial registration: NCT04047264

Study protocol: The full protocol is available at the request of the corresponding author.

Data collection: Data were collected intra-operatively at the Mayo Clinic in Rochester, MN from patients undergoing a planned neurosurgical resection of a known or suspected high-grade glioma. Data collection has been ongoing since early 2020; the last collection for this study was in the middle of 2022.

Outcomes: The primary outcome of the study was initially to determine the safety and feasibility of intra-operative microdialysis. Once this had been demonstrated in an initial set of patients, the primary outcome of the study was then to identify biomarkers of in situ gliomas across a diverse patient cohort using intra-operative microdialysis to sample extracellular metabolites. Secondary outcomes include:

- Evaluate the yield and specificity of microdialysate D-2HG as a candidate tumor biomarker to differentiate between IDH-mutated and IDH-wildtype gliomas.
- Identify biomarkers of tumor-associated processes including brain edema, brain infiltration with non-enhancing tumor, and tumor-associated hypoxia or necrosis.
- Determine the contribution of blood-brain barrier disruption to metabolite abundance within enhancing gliomas.
